# Molecular typing and drug sensitivity profiles of *M. Tuberculosis* isolated from refugees residing in Ethiopia

**DOI:** 10.1016/j.jctube.2023.100371

**Published:** 2023-04-15

**Authors:** Abyot Meaza, Getu Diriba, Musse Girma, Ammanuel Wondimu, Getnet Worku, Girmay Medhin, Gobena Ameni, Balako Gumi

**Affiliations:** a Aklilu Lemma Institute of Pathobiology (ALIPB), Addis Ababa University (AAU), P.O. Box 1176, Addis Ababa, Ethiopia; b Ethiopian Public Health Institute (EPHI), PO Box 1242, Swaziland Street, Addis Ababa, Ethiopia; c Department of Medical Laboratory Science, College of Medicine and Health Sciences, Jigjiga University, Ethiopia; d Department of Veterinary Medicine, College of Agriculture and Veterinary Medicine, United Arab Emirates University, PO Box 15551, Al Ain, United Arab Emirates

**Keywords:** Tuberculosis, Drug susceptibility testing, Spoligotyping, *M. tuberculosis*, Refugees

## Abstract

**Background:**

Refugees in developing countries have poor access to Tuberculosis (TB) care and control services. The understanding of genetic diversity and drug sensitivity patterns of *M. tuberculosis* (MTB) is important for the TB control program. However, there is no evidence that shows the drug sensitivity profiles and genetic diversity of MTB circulating among refugees residing in Ethiopia. This study aimed to investigate the genetic diversity of MTB strains and lineages, and to identify the drug sensitivity profiles of MTB isolated from refugees residing in Ethiopia.

**Methods:**

A cross-sectional study was conducted among 68 MTB positive cases isolated from presumptive TB refugees from February to August 2021. Data and samples were collected in the refugee camp clinics and both rapid TB Ag detection and region of difference (RD)-9 deletion typing were used to confirm the MTBs. Drug susceptibility test (DST) and molecular typing were done using Mycobacterium Growth Indicator Tube (MGIT) method and spoligotyping respectively.

**Results:**

DST and spoligotyping results were available for all 68 isolates. The isolates were grouped into 25 spoligotype patterns, which consisted of 1–31 isolates with 36.8% strain diversity. The international shared type (SIT)25 was predominant spoligotype pattern consisting of 31 (45.6%) isolates, followed by SIT24 comprising 5 (7.4%) isolates. Further investigation showed that 64.7% (44/68) of the isolates were belonged to CAS1-Delhi family and 75% (51/68) of the isolates were belonged to lineage(L)-3. Multi-drug resistance (MDR)-TB was observed only in one isolate (1.5%) for first-line anti-TB drugs and the highest level of mono-resistance, 5.9% (4/68), was observed for PZA(Pyrazinamide). Mono-resistance was observed in 2.9 % (2/68) and while 97.0% (66/68) of the MTB positive cases were susceptible to the second-line anti-TB drugs.

**Conclusion:**

The findings are useful evidence for the TB screening, treatment and control in refugee populations and surrounding communities in Ethiopia.

## Highlights

• Genetic diversity and DST of MTB can contribute for better TB control program.
• Spoligotyping and DST were conducted on MTB isolates obtained from refugees.
• 25 SITs identified, which consisted of 1–31 isolates with 36.8% strain diversity.
• Predominant SIT, family & lineage were SIT25, CAS1-Delhi, & Lineage-3 respectively.
• Most MTB strains (98.5%) were susceptible to both rifampicin and isoniazid.

## 1 Introduction

Tuberculosis is a major cause of ill health and one of the leading causes of death worldwide. Until the coronavirus pandemic, TB was the leading cause of death from a single infectious agent. An estimated 10.6 million people fell ill with TB worldwide in 2021 [1]. Drug resistant-TB (DR-TB) continues to be a public health threat. Resistance to rifampicin (RIF) and isoniazid (INH) is defined as MDR-TB. Resistance to RIF, the most effective first-line drug is of greatest concern because of MDR-TB. In 2021, the estimated proportion of people with TB who had MDR/RR-TB was 3.6% among new cases and 18% among those previously treat. Both MDR-TB and rifampicin-resistant (RR)-TB require second-line anti-TB treatment [1], [2].

In Ethiopia, TB is one of the major public health problems with annual estimated TB incidence of 132 cases per 100,000 population in 2021 [1]. The prevalence of MDR-TB prevalence was 1.03% among new and 6.52% among previously treated TB patients [3]. Although Ethiopia is transitioned out from the list of 30 high MDR/RR-TB burden countries, it is still among the 30 high TB burden countries [1].

In a systematic review of spoligotyping-based genetic diversity of MTB in Ethiopia [4], the predominant lineage was Euro-American (64.8%) followed by East African-Indian (23.0%) and T (48.0%) and CAS (3.0%) were identified as the top two clades. Furthermore, the three predominant shared types (spoligotype patterns) were SIT149, SIT53, and SIT25, each consisting of 420, 343, and 266 isolates, respectively, while, 15% of the strains were orphan [4].

To our knowledge, there is no literature that shows drug sensitivity profiles and genetic diversity of MTB circulating among refugees residing in Ethiopia. Due to the diverse and highly mobile nature of refugees, the existing national TB survey and surveillance mechanisms in Ethiopia often excludes the refugee population. Hence, this study aimed to investigate the genetic diversity of MTB and to identify the drug sensitivity profiles of MTB isolated from refugees residing in Ethiopia.

## 2 Materials and Methods

### 2.1 Study design

This study is a continuation of the previous study [5] on presumptive TB refugees residing in 12 refugee camps of Ethiopia from February 23, 2021 to August 25, 2021. A cross-sectional study was conducted on culture-positive MTB isolates obtained from the previous study [5]. The sample size of the previous study [5] was estimated based on the minimum required sample size for prevalence studies [6] and the total sample size for prevalence study was 610. The total sample size was allocated to the four-refugee camp complex of country of origin (South Sudan, Sudan, Somalia, Eritrea) based on their population size in the camp using proportional allocation method. Thus 107, 147, 324, and 32 participants were allocated for refugees originated from Eritrea, Somalia, South Sudan and Sudan, respectively. Seventy-one samples were culture positive. Sixty-eight MTB isolates that had pure MTB culture growths on LJ subculture; and were confirmed by both rapid TB Ag detection and RD-9 deletion typing were included in the current study whereas three isolates excluded from the study due to smooth and non-visible growth characteristics and not fulfilling the above inclusion criteria. First-line and second-line phenotypic DST and spoligotyping were done for 68 confirmed MTB isolates at the national TB reference laboratory, EPHI.

### 2.2 Study population

Refugees originated from the four neighboring countries of Ethiopia, namely, South Sudan, Sudan, Eritrea, and Somalia were the source of population. All confirmed TB cases in the selected refugee camp TB clinics during the study period and fulfill the eligibility criteria were the study population.

### 2.3 Drug susceptibility testing

DST was performed using the MGIT method [7]. The first-line anti-TB drugs were Streptomycin (SM), INH, RIF, Ethambutol (EMB) and Pyrazinamide (PZA). The critical concentration of the first line anti-TB drugs for MGIT was used as 1.0, 0.1, 1.0, 5.0, and 100 µg/ml for SM, INH, RIF, EMB, and PZA respectively [7]. The second-line anti-TB drugs Fluoroquinolones such as Clofazimine (CLO), Levofloxacin (LEV), Linezolid (LZD), and Ofloxacin (OFL) and injectables such as Amikacin (AMK), Capreomycin (CAP), and Kanamycin (KAN) susceptibility testing was done. 1.0 μg/mL of AMK, 2.5 μg/mL of CAP, 2.5 μg/mL of KAN, 1.0 μg/mL of LEV, 1.0 μg/mL of LZD, 1.0 μg/mL of CLO, and 2.0 μg/mL of OFL were used as the critical concentration of second line anti-TB drugs for MGIT method [7] based on the WHO standard [8]. Two MGIT tubes were inoculated with the test culture. A known concentration of a test drug was added to one of the MGIT tubes, and growth is compared with the MGIT tube without the drug (growth control). If the test drug was active against the isolated mycobacteria, it would inhibit the growth, resulting in suppression of fluorescence while the growth control would not be inhibited and would have increased fluorescence. Growth was monitored by MGIT 960 instrument which automatically interpreted results as susceptible or resistant. One H37RV sensitivity strain was run per batch of DST set for quality control purposes [7], [9].

### 2.4 Molecular typing

The RD-9 polymerase chain reaction (PCR) deletion typing was performed on heat-killed cells to confirm the presence or absence of RD9 to identify MTB from the other species of the *M. tuberculosis complex* as previously described by Brosch et al [10]. Three RD-9 primers: RD9flankF, RD9IntR and RD9flankR were used to identify isolates. The PCR amplification was performed in reaction mixture consisting of HotStarTaq Master Mix, distilled water, primers and DNA template (heat killed). The reaction was heated for 10 min at 95 °C for enzyme activation followed by 35 cycles of 1 min of denaturation at 95 °C, 0.5 min of annealing at 61 °C and 2 min of extension at 72 °C, and then a final extension at 72 °C for 10 min. Thereafter the product was removed from the thermocycler and run-on agarose gel electrophoresis. Detection of a band size of 396 bp was considered positive for *M. tuberculosis*, whereas detection of a band size of 575 bp was considered to be positive for the other members of the *M. tuberculosis complex* species (*M. bovis or M.africanum)* [11].

Confirmed MTB isolates were identified by spoligotyping as previously described by Kamerbeek et al [12] using a commercially available membrane following the manufacturer’s instructions (Mapmygenome, India). The direct repeat (DR) region was amplified by PCR using oligonucleotide primers DRa and DRb. DNA from known strains of M. bovis SB 1176 and H37Rv were used as positive controls, whereas Qiagen water was used as a negative control. A reaction mixture was prepared, consisting of Hot StarTaq Master Mix, primers, heat-killed cells, and distilled water. The mixture was heated for 15 min at 96 °C and then subjected to 30 cycles of 1 min at 96 °C, 1 min at 55 °C and 30 s at 72 °C. The amplified product was hybridized to a set of 43 immobilized oligonucleotides, each corresponding to one of the unique spacer DNA sequences within the DR locus. After hybridization, the membrane was washed twice in 2 × SSPE and 0.5% SDS and then incubated in streptavidin-peroxidase (HotStar, Crawley, UK) for 45–60 min at 42 °C. After hybridization, the DNA was detected by enhanced chemiluminescence and by exposure to X-ray film as specified by the manufacturer. The hybridization patterns were converted into binary and octal formats and compared with previously reported strains in the recent SITVIT2 database [12].

## 3 Data processing and statistical analysis

Computerized data was exported to STATA statistical software version 14 for data checking, cleaning, and descriptive analysis to fit the logistic regression model. Descriptive statistics were used to summarize frequencies, and percentages and presented in tables as appropriate. The genetic diversity and spoligotyping patterns were expressed as numbers, percentages and proportions. A web-based spoligotype databases such as SITVIT2 [13] and TB insight [14] were utilized to assign SITs, sub-lineages and major lineages for the isolates. The drug sensitivity profiles such as the level of mono/poly resistance for anti-TB drugs and the proportion of MDR/Pre-extensively drug resistance (XDR)/XDR-TB were expressed as numbers, frequencies, and percentages.

## 4 Results

### 4.1 Socio-demographic and clinical characteristics of MTB isolates

The country of origin of the refugees for MTB isolates was South Sudan 53, 77.9%), Somalia (9, 13.2%), Eritrea (4, 5.9%), and Sudan (2, 2.9%). The majority of the recovered isolates were from males (45, 66.2%), and the mean age of the participants was 33.4 years with a standard error of 13.8. The participants with previous TB treatment, TB contacts and HIV positive were 11.8%, 22.1%, and 11.8%, respectively. Twenty-two (32.4%) participants were current smokers and only two participants (2.9%) had a history of incarceration. Fourteen participants (20.6%) had no window in their tents and the mean number of households was 5.6 (Table 1).

**Table 1:** Socio-demographic and clinical characteristics of 68 confirmed MTBs isolated from presumptive TB refugees residing in refugee camps of Ethiopia.

Socio-demographic Characteristics	Category	Frequency	Percentage
Country of origin			
	Eritrea	4	5.9
	Somalia	9	13.2
	South Sudan	53	77.9
	Sudan	2	2.9
Age			
	12–18	4	5.9
	19–38	43	63.2
	39–58	15	22.1
	>58	6	8.8
Sex			
	Male	45	66.2
	Female	23	33.8
TB Classification			
	Newly diagnosed	60	88.2
	Previously treated	8	11.8
HIV status			
	Negative	48	70.6
	Positive	8	11.8
	Unknown	12	17.6
TB contact			
	TB contacts	15	22.1
	No TB contacts	53	77.9
Current smoker			
	Smoker	22	32.4
	Non-smoker	46	67.6
Availability of Window			
	Window	54	79.4
	No window	14	20.6
History of incarceration			
	Incarcerated	2	2.9
	Not incarcerated	66	97.1
No of household			
	1–5	36	52.9
	6–10	31	45.6
	>10	1	1.5

### 4.2 Molecular typing of MTB isolates

All 68 isolates were confirmed as MTB by the rapid TB Antigen test and RD9-based PCR. Spoligotyping of these isolates identified 25 spoligotype patterns consisting of 1–31 isolates, thus spoligotyping revealed higher diversity (36.8 %). Among 25 spoligotype patterns, 16 were singlets and nine were clustered. The predominant spoligotype pattern was SIT25 consisting of 31 (45.6%) isolates, followed by SIT24 consisting of 5(7.4%) isolates. Four (5.9%) isolates were orphans (Table 2 and Supplementary material 1).

**Table 2:** Distribution of SIT, Sub-lineage, and Lineage for 68 MTB isolates from Spoligotyping.

**Octal**	**SP**	**Family/Sub-lineage**	**Major Lineage**	**SP Frequency n (%)**	**Binary Code**
703777740003171	25	CAS1-Delhi	Lineage 3	31 (45.6)	
703777740003031	24	CAS1-Delhi	Lineage 3	5 (7.4)	
703777700003771	142	CAS1-Delhi	Lineage 3	3 (4.4)	
703777700003171	2392	CAS	Lineage 3	3 (4.4)	
703777740003771	26	CAS1-Delhi	Lineage 3	2 (2.9)	
777737777760771	37	T3	Lineage 4	2 (2.9)	
577777002060771	788	Unknown	Lineage 4	2 (2.9)	
703737740003171	1198	CAS1-Delhi	Lineage 3	2 (2.9)	
703777747777771	1200	Unknown	Lineage 1	2 (2.9)	
477777277413771	10	EAI8-MDG	Lineage 1	1 (1.5)	
703377400001771	21	CAS1-Kili	Lineage 3	1 (1.5)	
777777777720771	50	H3	Lineage 4	1 (1.5)	
777777777760771	53	T1	Lineage 4	1 (1.5)	
777777777760601	244	T1	Lineage 4	1 (1.5)	
777756777760771	302	X1	Lineage 4	1 (1.5)	
777737747413771	726	EAI6-BGD1	Lineage 1	1 (1.5)	
777777777420771	777	Ural-1	Lineage 4	1 (1.5)	
777777403760771	1688	T1	Lineage 4	1 (1.5)	
503767740003171	1945	CAS1-Delhi	Lineage 3	1 (1.5)	
777773777720771	2089	H3	Lineage 4	1 (1.5)	
000000000000000	2669	ATYPIC	Lineage 2	1 (1.5)	
600001740003171	Orphan	Unknown	Lineage 3	1 (1.5)	
703767600001771	Orphan	Unknown	Lineage 3	1 (1.5)	
703737700003771	Orphan	Unknown	Lineage 3	1 (1.5)	
772701002060771	Orphan	Unknown	Lineage 4	1 (1.5)	

SP-Spoligotyping Pattern.

**Fig. 1:**
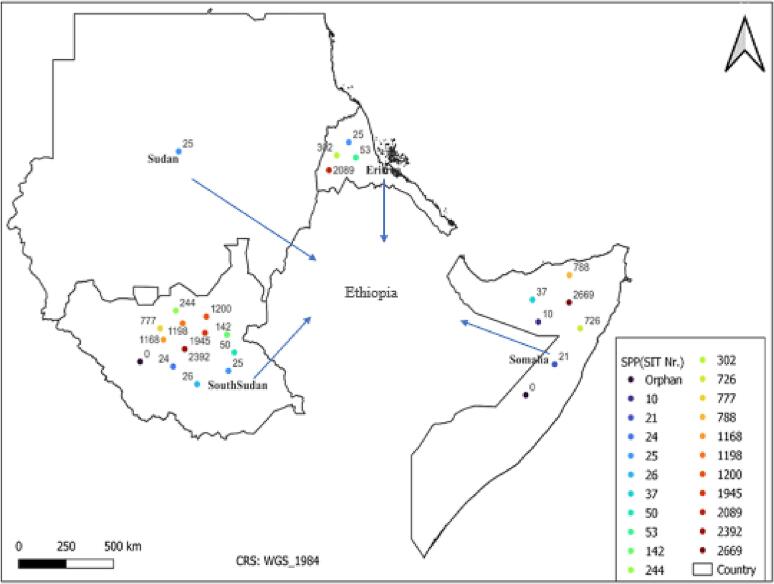
Types of MTB spoligotyping patterns (SIT numbers) identified in the study and their origin of countries. The four arrows indicate the influx of the refugees from Eritrea, Somalia, South Sudan and Sudan to Ethiopia. SPP-spoligotyping pattern, SIT Nr. — International shared type number.

The source of the MTB spoligoyping patterns were originated from four neighboring countries of Ethiopia, namely, Eritrea, Somalia, South Sudan and Sudan. SIT25 was predominant among South Sudan and Sudan origin; and variable MTB spoligotyping patterns were identified from Somalia and Eritrea (Fig. 1 and Supplementary material 1).

**Fig. 2:**
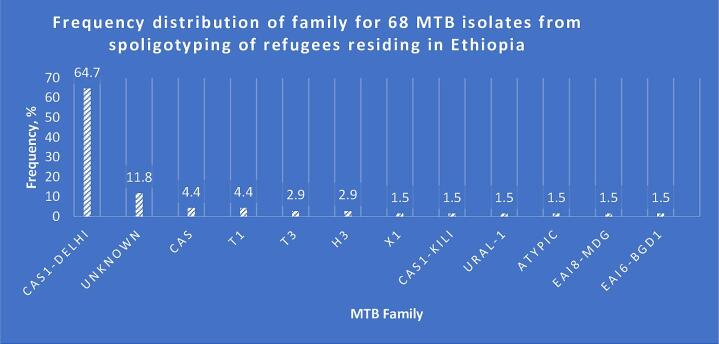
Frequency distribution of family for 68 MTB isolates from Spoligotyping.

Further investigation of the spoligotype patterns into family and lineage using SITVIT2 and TB insight databases showed that the majority, 64.7% (44/68) of the isolates belonged to the CAS1-Delhi family. The CAS1-Delhi was the most predominant family from South Sudanese (41/53) and Sudanese (2/2) isolates whereas variable families or sub-lineages were identified from Somalia (T3, CAS1-Kili, EAI8-MDG, EAI8-MDG) and Eritrean isolates (T1, X1, H3, CAS1-Delhi). Among nine Somali isolates, three isolates were identified as unknown family. The most frequent lineage was L3 (51 isolates, 75%), followed by L4 (13 isolates, 19.1 %) (Fig. 2 and Supplementary material 1).

### 4.3 Drug sensitivity profiles of MTB isolates

DST results were valid and available for all 68 isolates. Mono-resistance to any one of the five first-line ant-TB drugs (SM, INH, RIF, EMB, and PZA) was observed in 11.8% (8/68) of the cases. The highest level of mono-resistance, 5.9% (4/68), was observed for PZA among new cases, strains belonging to the L3. Moreover, MDR-TB was detected in 1.5% (1/68) of the isolates while, 86.7% (59/68) of the isolates were susceptible to all of the first-line anti-TB drugs (Table 3).

**Table 3:** First-line and second-line phenotypic drug sensitivity profiles among 68 MTB isolates.

First line DST Profile, n (%)				Second-line DST Profile, n (%)			
Drugs	New cases (n = 60)	Prev. treated (n = 8)	Total (n = 68)	Drugs	New cases (n = 60)	Prev. treated (n = 8)	Total (n = 68)
Susceptible							
SM + INH + RIF + EMB + PZA	53 (88.3)	6 (75)	59 (86.7)	AMK + CAP + KAN + OFL + LEV + LZD + CLO	58 (96.6)	8 (100)	66 (97.0)
Resistance							
*Mono-resistance*							
SM	1 (1.7)	1 (12.5)	2 (2.9)	KAN	1 (1.7)	0	1 (1.5)
INH	0	1 (12.5)	1 (1.5)	CLO	1 (1.7)	0	1 (1.5)
RIF	0	0	0				
EMB	1 (1.7)	0	1(1.5)				
PZA	4 (6.6)	0	4 (5.9)				
*MDR*							
INH + RIF + EMB	1 (1.7)	0	1 (1.5)				
Total	60 (100)	8 (100)	68 (100)		60 (100)	8 (100)	68 (100)

Prev-previously treated cases; S-Susceptible, R-Resistance; first-line anti-TB drugs: EMB-Ethambutol, INH-Isoniazid, PZA-Pyrazinamide, RIF-Rifampicin, SM-Streptomycin; second-line anti-TB drugs: AMK-Amikacin, CAP-Capreomycin, CLO-Clofazimine, KAN-Kanamycin, LEV- Levofloxacin, LZD-Linezolid, OFL-Ofloxacin.

Mono-resistance was observed in 2.9% (2/68) of the cases for second-line anti-TB drugs (CLO, and KAN) while 97.0% (66/68) of the MTB positive cases were susceptible to all of the second-line anti-TB drugs (Fluoroquinolones: CLO, LEV, LZD, & OFL and injectables: AMK, CAP, & KAN). There was no detected pre-XDR-TB or XDR-TB (Table 3).

## 5 Discussions

To our knowledge this is the first study in Ethiopia, where nearly 800, 000 refugees sheltered [15], investigating the molecular typing and the drug sensitivity profiles of MTB among refugees. In this study, the predominant spoligotyping pattern, family, and lineage were SIT25, CAS1-Delhi, and L3, respectively. High diversity of spoligotyping patterns (36.8%) was found and grouped into 25 clusters. Moreover, MDR-TB was detected in 1.5% of the isolates while, 86.8% of the isolates were susceptible to all of the first-line anti-TB drugs. Mono-resistance was observed in 2.9% of the cases for second-line anti-TB drugs while 97.1% of the MTB positive cases were susceptible to all of the second-line anti-TB drugs.

Although the emerging high-throughput technologies such as whole genome sequencing (WGS) is better in defining MTB strain diversity and has higher discriminatory power [16], [17], molecular typing remains a useful molecular technique for characterization of MTB genotypes and in defining MTB strain diversity [18], [19]. Additionally, since there was concordance between WGS and spoligotyping results [17], we consider that spoligotyping is still an efficient and valid technique for genotyping MTB in developing countries, where resources for higher technology are scarce.

Our study reported a comparable strain diversity compared to a study [20] in the Somali region of Ethiopia which reported strain diversity of 22% (71/323). This could be similar MTB family/clades might be shared to both population due to the common geographical area of the study population in which Somalis in Somali region of Ethiopia live as surrounding community of the Somali refugees. This will highlight the need for strong intervention in screening and surveillance of TB and other infectious diseases on refugees before movement of refugees to the community and vice versa. In another study [21] in Sudan higher strain diversity 95% (112/118) was reported compared to our study. The higher diversity of MTB strains reported in the previous study [21] might be due to the high mobility of migrants and refugees that stayed in the study setting for temporary settlement. The additional reason could be the transmission of TB in the area might resulted for the dominancy of the strain in the study area.

The predominant spoligotyping pattern, family and lineage in the current study were different from the previously reported findings in Ethiopia [4]. In the systematic review of 21 studies in Ethiopia [4], the predominant spoligotyping pattern was SIT149 in the Ethiopian population; while SIT25 was reported as the predominant spoligotyping pattern in this study. The predominant family and lineage of MTB were T family and L4 in the Ethiopian population while CAS1-Delhi family and L3 found to be predominant in the refugees of this study. The predominant strain, family, and lineages difference could be influenced by the country of origin of the refugees and simillar findings were also reported from the geographical locations where the study population were originated [21], [22]. The findings have public health implications in providing the responsible stains for TB epidemic to the TB control program for necessary TB control planning and interventions in refugees and surrounding community in Ethiopia.

Currently, Ethiopia is among the 30 high TB burden countries whereas Somalia is among the 30 high MDR/RR-TB burden countries [1]. The yield observed in this study was lower compared to similar studies among refugees [23], [24], [25], [26]. The lower yield in our finding could be explained by the study method used in which the passive prevalence method was undertaken to enroll the presumptive refugees in the study while active case finding [27] and enhanced screening method [25] were used to enroll the study participants that might increases the detection of TB and MDR-TB cases. The other reason could be that the number of MTB positive cases were higher, 264 MTB positive cases and 241 MTB positive cases [24] compared to the current study which is 68 MTB positive cases. However, the lower proportion of MDR-TB in the current study could be encouraging in the TB control effort in the refugee population which need further evidence by the implementation of routine TB drug resistance surveillance in the refugee population.

There was neither pre-XDR-TB nor XDR-TB detected in the current study and a similar result was reported by a study [28] from the Somali region of Ethiopia where no pre-XDR/XDR-TB was reported. In support of these, a lower prevalence of pre-XDR-TB and no XDR-TB report [29] detected from Ethiopian national routine laboratory-based DR surveillance. Despite the low prevalence of pre-XDR-TB and no XDR-TB report from the national routine surveillance [29] and no pre-XDR/XDR-TB from our study, contact investigation, specimen referral system and access to rapid TB diagnosis testing should be improved for better detection of DR-TB in Ethiopia. However, the drug resistance survey in Tibetan refugee camps in India had found three refugees with XDR-TB [23]. This might be due to overcrowded living conditions of Tibetan refugees of whom more than half of TB cases in these refugees occurred in congregated settings. Moreover, use of active case finding in the study might increase the detection of undiagnosed XDR-TB. These findings have also public health implications in monitoring the status of DR-TB in refugee population and providing evidence to strengthen the TB control activities across the cross-border area of neighboring countries with Ethiopia.

The limitation of this study was the use of the classical spoligotyping method which has limited discriminatory power for classification of SITs and lineages compared to Mycobacterial Interspersed Repetitive Unit-Variable Number Tandem Repeat (MIRU-VNTR) and WGS. Moreover, the technique is likely prone to homoplasy which might have an effect on the proportion of isolates clustering.

## 6 Conclusion

The identification of predominant SIT25, CAS1-Delhi, and lineage 3, in the refugees could provide new insight in molecular epidemiology of MTB in refugee population and surrounding community in Ethiopia. In our study, Most MTB strains (98.5%) were susceptible to both rifampicin and isoniazid., which could encourage the TB control program. Thus, these findings are useful evidence for the TB screening and control in refugees and surrounding communities in Ethiopia. Moreover, further TB transmission studies and active TB drug resistance surveillance are recommended in the refugee camps of Ethiopia.

## 7 Contributors

AM designed the study; BG oversaw the study; AM coordinated the study sites. Statistical analysis was undertaken by AM, and reviewed by GM. AM, GD, MG, AW, and GW contributed to the data collection and laboratory investigations. The manuscript draft was developed by AM with input from GM, GA and BG. All authors contributed editing the draft manuscript and approval of the final version of the manuscript.

## Funding source

This research did not receive any specific grant from funding agencies in the public, commercial, or not-for-profit sectors.

## Ethical approval statement

The study was approved by the ethical review committee of AAU and EPHI. Written informed consent or assent was obtained from each study participant before data and sample collection. Patients’ names or IDs were confidential during the study procedure.

## Declaration of Competing Interest

The authors declare that they have no known competing financial interests or personal relationships that could have appeared to influence the work reported in this paper.

## References

[1] World Health Organization Global Tuberculosis Report 2022 2022 Geneva [Acessed on 01 October 2022].

[2] World Health Organization Global tuberculosis report 2020 2020 Geneva [Acessed on 18 September 2021].

[3] Ministry of Health, Ethiopia. Annual Performance Report, 2014 EFY/2021-2022. Ministry of Health, Ethiopia 2022. [Acessed on 02 October 2022].

[4] Tulu B., Ameni G. (2018). Spoligotyping based genetic diversity of Mycobacterium tuberculosis in Ethiopia: a systematic review. BMC Infect Dis.

[5] Abyot Meaza, Bazezew Yenew, Miskir Amare, Ayinalem Alemu, Michael Hailu, Dinka Fikadu Gamtesa , Mirgissa Kaba, Girmay Medhin, Gobena Ameni, and Balako Gumi. Bacteriologically confirmed Plumonary Tuberculosis and associated factors among presumptive Tuberculosis refugees residing in refugee camps of Ethiopia. 2022. [Unpublished data].

[6] Naing L., Winn T., Rusli B. (2006). Practical issues in calculating the sample size for prevalence studies. Arch Orofac Sci.

[7] Siddiqi S.H.G.S. (2006).

[8] World Health Organization. Technical report on critical concentrations for drug susceptibility testing of medicines used in the treatment of drug-resistant tuberculosis. WHO; 2018.

[9] Kent PT, K. P. Public Health Mycobacteriology a guide for the level III laboratory. Atlanta, CDC 1985. [Acessed on 18 September 2021].

[10] Brosch R, Gordon SV, Marmiesse M, Brodin P, Buchrieser C, Eiglmeier K, et al. A new evolutionary scenario for the Mycobacterium tuberculosis complex. Proceedings of the national academy of Sciences. 2002;99(6):3684-9. 10.1073/pnas.052548299 PMC122584 11891304

[11] Parsons L.M., Brosch R., Cole S.T., Ak S., Loder A., Bretzel G. (2002). Rapid and simple approach for identification of Mycobacterium tuberculosis complex isolates by PCR-based genomic deletion analysis. J Clin Microbiol.

[12] Kamerbeek J., Schouls L., Kolk A., Van Agterveld M., Van Soolingen D., Kuijper S. (1997). Simultaneous detection and strain differentiation of Mycobacterium tuberculosis for diagnosis and epidemiology. J Clin Microbiol.

[13] Couvin D., David A., Zozio T., Rastogi N. (2019). Macro-geographical specificities of the prevailing tuberculosis epidemic as seen through SITVIT2, an updated version of the Mycobacterium tuberculosis genotyping database. Infect Genet Evol.

[14] Shabbeer A., Cowan L.S., Ozcaglar C., Rastogi N., Vandenberg S.L., Yener B. (2012). TB-Lineage: an online tool for classification and analysis of strains of Mycobacterium tuberculosis complex. Infect Genet Evol.

[15] United Nation Humaniterian Comission for Refugee. Ehiopia Fact sheet. UNHCR, September 2018.

[16] World Health Organization. The use of next-generation sequencing technologies for the detection of mutations associated with drug resistance in Mycobacterium tuberculosis complex: technical guide. WHO; 2018.

[17] Perea Razo C.A., Rodriguez Hernandez E., Ponce S.I.R., Milian Suazo F., Robbe-Austerman S., Stuber T. (2018). Molecular epidemiology of cattle tuberculosis in Mexico through whole-genome sequencing and spoligotyping. PLoS One.

[18] Barnes P.F., Cave M.D. (2003). Molecular epidemiology of tuberculosis. N Engl J Med.

[19] Ei P.W., Aung W.W., Lee J.S., Choi G.-E., Chang C.L. (2016). Molecular strain typing of Mycobacterium tuberculosis: a review of frequently used methods. J Korean Med Sci.

[20] Worku G., Gumi B., Mohammedbirhan B., Girma M., Sileshi H., Hailu M. (2022). Molecular epidemiology of tuberculosis in the Somali region, eastern Ethiopia. Front Med.

[21] Elegail A., Mohamed N.Y.I., Nour E.O.M., Hoffner S., Haile M. (2018). Molecular characterization of Mycobacterium tuberculosis isolates from pulmonary tuberculosis patients in Khartoum, Sudan. Int J Mycobacteriology.

[22] Eldirdery M.M., Alrayah I.E., ElkareIm M.O.A., Khalid F.A. (2015). Genotyping of pulmonary Mycobacterium tuberculosis isolates from Sudan using spoligotyping. Am J Microbiological Res.

[23] Salvo F., Dorjee K., Dierberg K., Cronin W., Sadutshang T., Migliori G. (2014). Survey of tuberculosis drug resistance among Tibetan refugees in India. Int J Tuberc Lung Dis.

[24] Githui W., Hawken M., Juma E., Godfrey-Faussett P., Swai O., Kibuga D. (2000). Surveillance of drug-resistant tuberculosis and molecular evaluation of transmission of resistant strains in refugee and non-refugee populations in North-Eastern Kenya. Int J Tuberc Lung Dis.

[25] Oeltmann J.E., Varma J.K., Ortega L., Liu Y., O’Rourke T., Cano M. (2008). Multidrug-resistant tuberculosis outbreak among US-bound Hmong refugees, Thailand, 2005. Emerg Infect Dis.

[26] Ministry of Health, Uganda. Ugandan National Tuberculosis and Leprosy Division Report. Report 2018. [Acessed on 12 August 2021].

[27] Dierberg K.L., Dorjee K., Salvo F., Cronin W.A., Cirillo D., Sadutshang T. (2016). Improved detection of tuberculosis and multidrug-resistant tuberculosis among Tibetan refugees, India. Emerg Infect Dis.

[28] Worku G., Gumi B., Girma M., Mohammedbirhan B., Diriba G., Seid G. (2022). Drug sensitivity of clinical isolates of Mycobacterium tuberculosis and its association with bacterial genotype in the Somali region. Eastern Ethiopia Front Public Health.

[29] Diriba G., Alemu A., Tola H.H., Yenew B., Amare M., Eshetu K. (2022). Pre-extensively drug-resistant tuberculosis among multidrug-resistant tuberculosis patients in Ethiopia: a laboratory-based surveillance study. IJID Regions.

